# The Life-Threatening Risk of a Dirty Wound: A Lesson From the Past

**DOI:** 10.7759/cureus.9967

**Published:** 2020-08-23

**Authors:** Mohammad Zaid Seegoolam, Muhammad Hafiz Kamarul Bahrin, Kayteck Ling, Altaf Palejwala

**Affiliations:** 1 Internal Medicine, Queen's Hospital Burton, Burton-On-Trent, GBR; 2 Geriatrics Medicine, Queen's Hospital Burton, Burton-On-Trent, GBR; 3 Gastroenterology, Queen's Hospital Burton, Burton-On-Trent, GBR

**Keywords:** tetanus, vaccination, tetanus severity score (tss), notifiable disease, tetanus immunoglobulin

## Abstract

Tetanus is a severe and potentially life-threatening infection caused by the bacterium Clostridium Tetani. It is a gram-negative anaerobe, often found in soil in spore form and in the gastrointestinal tract of humans and animals. It produces a potent neurotoxin called tetanospasmin. The presence of this toxin on the affected wound contributes to its pathogenesis. In developed countries such as the United Kingdom, tetanus poses a diagnostic challenge as cases are becoming scarce and, therefore, difficult to diagnose in an acute setting following the national immunisation programme in 1961. The prognosis of an acute tetanus can be derived from several risk-stratifying scoring systems such as the Tetanus Severity Score (TSS), with any score above 8 representing a 53% case-fatality rate. Prompt clinical diagnosis, immediate delivery of treatment and strict adherence to the national vaccination programme are paramount to suppress the incidence and the fatality rate from tetanus.

## Introduction

Tetanus is a severe and potentially life-threatening infection caused by the bacterium Clostridium Tetani. It is a gram-negative anaerobe, often found in soil in spore form and in the gastrointestinal tract of humans and animals [1]. It produces a potent neurotoxin called tetanospasmin [1]. The presence of this toxin on the affected wound contributes to its pathogenesis. In the United Kingdom, tetanus infection has almost been eradicated following the national vaccination programme in 1961; hence, its incidence nowadays is scarce. Tetanus infection is classified into generalised tetanus, localised tetanus, and cephalic tetanus [2], with the latter two being the rarer forms of the disease. We describe a case of an 87-year-old male patient who presented with painful muscular spasm, risus sardonicus, and dysphagia two weeks after sustaining dirty laceration on his palm following a mechanical fall. Immunisation history, unfortunately, was unclear as there was no available record in patient's possession. Blood tests, including adjusted calcium level, were unremarkable. Clinical diagnosis of generalised tetanus infection was made and managed with intramuscular immunoglobulin, antibiotics, benzodiazepines and surgical wound debridement.

## Case presentation

An 87-year-old male patient attended the ED on the 11th of April 2020 after he fell in his garden sustaining deep laceration on the lateral aspect of his right palm from rusty spikes on a metal railing. The size of this wound was unfortunately not recorded. He was unsure of his vaccination status in the last 10 years. After superficial wound cleansing and stitching, he was given a shot of tetanus toxoid vaccine and was discharged. The wound was dressed nicely using a simple non-adhesive wound dressing. Patient presented again to the emergency department after two weeks with acute onset locked jaw, dysphagia, risus sardonicus and generalised muscular rigidity, particularly affecting the neck. The locked jaw resulted in painful tongue ulceration, impairing his ability to eat or drink. The wound dressing appeared to be soaked with blood-stained yellowish discharge.

During this admission, his observation was stable with heart rate of 90 beats per minute, blood pressure of 110/70, temperature of 36.8-degree Celcius, respiratory rate of 16 breaths per minute and oxygen saturation level of 96% on 2 litres of oxygen. The blood test results were also unremarkable with an adjusted calcium level of 2.41 mmol/L (normal range: 2.20-2.62). The complete laboratory investigation results were as in Tables 1 and 2.

**Table 1:** Blood investigation result during the second attendance at the emergency department Note that CK was raised, indicating convulsing muscle tissues probably as a result of changes in muscle fibre membrane permeability due to the tetanospasmin toxin

Blood investigation	Value	Normal Range
Haemoglobin (Hb)	120	140–170 g/L
White cell count (WCC)	10.4	4.5–11 x 10^9^ cells/L
Platelets	352	150–350 x 10^9^/L
Sodium (Na)	133	136–145 mmol/L
Potassium (K)	4.7	3.5–5 mmol/L
Urea	7.1	8-21 mg/dL
Creatinine	110	61.9-115 µmol/L
Creatinine Kinase (CK)	1000	30-170 U/L
C-reactive protein (CRP)	17	<10 mg/L
Adjusted calcium	2.41	2.2–2.6 mmol/L
Magnesium	0.82	0.62–0.99 mmol/L

**Table 2:** Microbiology results of wound swabs taken following administration of Intravenous Immunoglobulin (IVIG)

	Organism	Growth level	Sensitivity
Organism 1	Staphylococcus Aureus	Moderate Growth	Erythromycin
			Flucloxacillin
Organism 2	Clostridium Perfringens	Moderate Growth	Metronidazole

**Figure 1:**
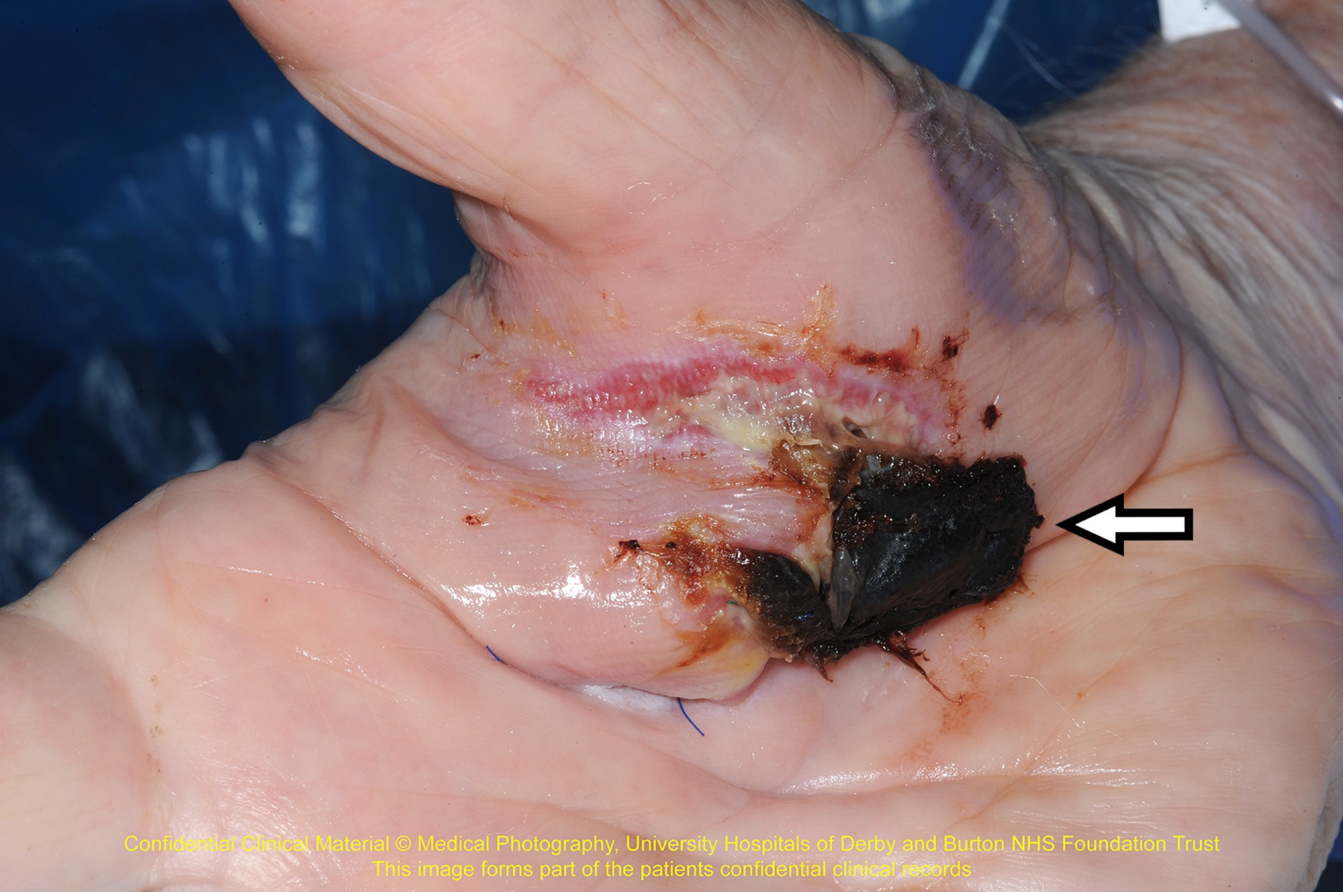
Wound appearance during the second attendance at the emergency department. Arrow shows necrotic tissue over the puncture wound

Physical examination of the wound revealed pus-discharging deep laceration with a big patch of necrotic tissue, about 3 cm in length in the lateral aspect of the right palm. There was also localised swelling over the affected area. Stitches from previous emergency department attendance were still intact. The wound is tender and warm on touch. Distally, neurovascular status was intact. The wound appearance was shown in Figure 1.

In the first instance, the diagnosis of tetanus was made. Subsequently, he was administered intramuscular tetanus immunoglobulin for two consecutive days; 250 units during the acute setting and after 24 hours of admission.

Serological study blood test and wound swabs were unfortunately only obtained after the immunoglobulin was given. Prompt microbiologist advice was sought, and the patient was then commenced on intravenous metronidazole and co-amoxiclav as recommended. Public Health England was notified. Patient was also given intravenous diazepam to stop the muscle spasm. Following the Trust guideline, intensive care unit review was sought in order to optimise patient’s supportive care. As no significant organ support was required, our patient was admitted into the medical ward overnight for close monitoring. He remained stable throughout his admission.

Orthopaedic surgical review the following day demonstrated an actively pus-discharging wound and urgent surgical debridement was thought to be the definitive treatment. Patient was then transferred to the regional major trauma center to undergo this surgery.

## Discussion

Tetanus is an infection caused by the bacterium clostridium tetani. It occurs when the spores of clostridium tetani gain access into damaged human tissue, where they transform into vegetative rod-shaped bacteria following inoculation [3]. In this state, they are capable of producing the metalloprotease tetanus neurotoxin, called tetanospasmin. The bacteria travel to the spinal cord and brainstem via retrograde axonal transport within the motor neuron and secretes its neurotoxin into the adjacent inhibitory interneurons subsequently blocking neurotransmission across the synaptic cleft. The net effect is inactivation of neurotransmission that modulates anterior horn cells and muscle contraction. The resulting loss of inhibition of anterior horn cells and autonomic neurons causes increased muscle tone, painful spasms, and widespread autonomic instability [4].

In a generalised tetanus, the neurotoxin disinhibition of neurotransmission impairs neural control of adrenal release of catecholamines. This subsequently results in a hypersympathetic state characterised by sweating, tachycardia, and hypertension. Generally, tetanus toxin produces a long-lasting effect and neuronal recovery requires the growth of new axonal nerve terminals [5].

Tetanus poses a diagnostic challenge as cases become increasingly rare following a successful national immunization programme, leading to delayed diagnosis in developed countries such as the UK. Between 2001 and 2014, only 96 cases were reported across the UK, with an annual average incidence of 0.13 per million [6]. According to Public Health England, it is primarily a clinical diagnosis characterised by acute illness with muscle spasms or hypertonia in the absence of other more likely diagnosis. Several severity scores have been developed to risk-stratify the prognosis and mortality outcome for patients with tetanus. Tetanus Severity Score (TSS) invented by Thwaites et al. in 2006 demonstrated relatively better sensitivity and specificity balance as compared to the traditional scoring systems namely the Dakar and the Phillips score. TSS value of 8 or more correlates with a 53% case-fatality rate [7].

In generalised tetanus, the key clinical features are trismus, painful muscle contractions of trunk muscles and generalised spasms, frequently associated with opisthotonus. As diagnosis is purely clinical, laboratory investigations only serve the purpose of supporting the diagnosis. This includes a panel of tetanus antibody detection and wound tissue Polymerase Chain Reaction test (PCR) and culture. Tetanus antibody detection of less than 0.1 U/ml indicates inadequate immunity hence is suggestive of the likelihood of its presence. Wound culture for c. tetani antigen, especially following immunoglobulin administration is associated with poor yield. However, treatment should not be delayed by investigation results [8].

The aim of treatment is to initially reduce the circulating bacterial load using intravenous antibiotics. Metronidazole is often used for this purpose versus benzylpenicillin despite the latter being traditionally used as the first line treatment. Metronidazole is associated with better outcome and lesser use of sedative to treat the resulting muscle spasm from tetanus, according to a study by Ahmadsyah et al. and Yen et al. [9,10]. The next step is to neutralise the toxic effect from the circulating unbound toxin using IVIG. The gold treatment standard is surgical debridement of the infected wound in order to eradicate spores and to alter conditions for bacteria germination [11].

Benzodiazepines are shown to be beneficial to reduce the painful muscle spasms due to tetanus. This could be administered alongside intravenous magnesium sulphate, which was shown to benefit those with respiratory compromise, as demonstrated by James [12]. Patients should be closely monitored preferably in an intensive care unit setting and supportive treatment such as mechanical ventilation to support airway compromise should be provided.

Another challenge in managing tetanus is that it does not confer immunity among those who were affected by it the first time and the effect of intravenous immunoglobulin is only temporary. Affected individuals may still contract life-threatening tetanus if exposed to it for the second time. Therefore, early vaccination with tetanus toxoid is paramount upon contracting the disease regardless of previous vaccination status in order to stimulate long-term humoral and cellular immunity [13]. Clinical evidence suggests that the toxoid vaccine should be injected at a different site from the immunoglobulin injection site to prevent its ‘neutralisation’ by passive immunity.

## Conclusions

The rarity of tetanus in developed countries such as the United Kingdom poses diagnostic and management challenges in the acute setting. The aim of treatment should always be about neutralising its toxin effect, eliminating the harbouring site of bacteria, reducing bacterial load and intensive supportive therapy. In the long run, vaccination programme should be adhered strictly by the general population as evidence for immunity acquisition following acute exposure is scarce, and herd immunity is not theoretically possible for tetanus. Our case report demonstrated that with good clinical acumen, a patient's disease course at the initial stage could be reversed.
